# Histopathological and Molecular Study of Pacific Oyster Tissues Provides Insights into *V. aestuarianus* Infection Related to Oyster Mortality

**DOI:** 10.3390/pathogens9060492

**Published:** 2020-06-20

**Authors:** Daniela Mandas, Fulvio Salati, Marta Polinas, Marina Antonella Sanna, Rosanna Zobba, Giovanni Pietro Burrai, Alberto Alberti, Elisabetta Antuofermo

**Affiliations:** 1 Fish Diseases and Aquaculture Center, IZS of Oristano, 09170 Oristano, Italy; danielamandas@gmail.com (D.M.); fulviosal@hotmail.com (F.S.); 2 Department of Veterinary Medicine, University of Sassari, 07100 Sassari, Italy; mpolinas@uniss.it (M.P.); msanna@uniss.it (M.A.S.); zobba@uniss.it (R.Z.); gburrai@uniss.it (G.P.B.); 3 Mediterranean Center for Disease Control (MCDC), University of Sassari, 07100 Sassari, Italy

**Keywords:** *Crassostrea gigas*, aquaculture, real time PCR, histology, Sardinia, bacteria, mass mortality

## Abstract

Consumer preference for healthy and sustainable food products has been steadily increasing in recent years. Bivalve mollusks satisfy these characteristics and have captured ever-increasing market shares. However, the expansion of molluscan culture in worldwide and global trade have favored the spread of pathogens around the world. Combined with environmental changes and intensive production systems this has contributed to the occurrence of mass mortality episodes, thus posing a threat to the production of different species, including the Pacific oyster *Crassotrea gigas*. In the San Teodoro lagoon, one of the most devoted lagoons to extensive Pacific oyster aquaculture in Sardinia, a mortality outbreak was observed with an estimated 80% final loss of animal production. A study combining cultural, biomolecular and histopathological methods was conducted: (1) to investigate the presence of different *Vibrio* species and OsHV-1 in selected oyster tissues (digestive gland, gills, and mantle); (2) to quantify *Vibrio aestuarianus* and to evaluate the severity of hemocyte infiltration in infected tissues; (3) to produce post-amplification data and evaluating ToxR gene as a target for phylogenetic analyses. Results provide new insights into *V. aestuarianus* infection related to oyster mortality outbreaks and pave the way to the development of tools for oyster management.

## 1. Introduction

The bivalve mollusks market has a significant economic impact worldwide and it represents about 15% of total aquaculture production. The Pacific oyster *Crassostrea gigas* has become one of the most commonly farmed mollusk species worldwide [1], with a production of approximately 5,600,000 tons in 2016 only [2]. In Italy, despite *C. gigas* representing less than 1% of the total mollusk production demand for oysters, demand is growing and continues to trend upward [3]. Italy represents the third-largest market in Europe [4]. The expansion of molluscan culture worldwide and global trade have favored the spread of pathogens around the world [5] which, combined with environmental changes and intensive production systems, contributed to the occurrence of mass mortality episodes thus posing a threat to the production of different species, including the Pacific oyster [6].

Mass mortality has occurred in farmed oysters for over five decades with great economic impact, with mortality reaching 90% to 100%, and non-specific initial symptoms, such as reduced growth and decreased feeding activity. An oysters physiological and environmental factors (water temperature and salinity), and presence of pathogens (virus and bacteria) have been implicated as potential risk factors for mass mortality [7,8,9,10].

Mortality outbreaks of oysters have been recorded in larvae and juvenile specimens and have been mostly attributed to a variant of type 1 oyster herpesvirus (OsHV-1 µvar). Adults can also be affected but they rarely show tissue damage [9,10,11,12]. Literature also reports that OsHV-1 µvar can persist for a long time in the infected hosts without apparent symptoms [13,14]. Thus, as highlighted by several authors, the detection of abnormalities in oyster organs is paramount to differentiate affected from asymptomatic oysters. 

Beside OsHV-1, several *Vibrio* species have been found to coinfect oysters during mortality episodes, although their role in disease is not yet completely understood and appears controversial [15]. Several *Vibrio* strains were isolated from healthy and moribund oysters, and numerous reports indicate *V. aestuarianus* as the main causative agent in adult oysters, sometimes in synergy with *V. splendidus* [16,17,18,19]. Nevertheless, data about *V. aestuarianus* and oyster interaction in the field are poorly reported in the literature, while most data derives from experimental challenges [6]. However, oyster mortality outbreaks and the presence of *V. aestuarianus* have been observed also in Italy [3,20].

As a general rule, the sole detection of *Vibrio* spp. in mollusks doesn’t necessarily relate to disease establishment and progression, for these microorganisms are ubiquitous in marine and brackish waters. Thus, combining the polymerase chain reaction (PCR) and histology approaches is necessary to determine the pathological significance of *Vibrio* in affected oysters. In fact, although PCR is the standard method for pathogen identification, histopathology represents an important screening tool in bivalve pathology providing crucial information on the presence and extent of hemocyte infiltration, and type and severity of tissue lesions associated with the presence of bacteria [5,6,21,22,23].

In general, fragmented information is available on the description of histological alterations in free-living (farmed) infected oysters, and only few studies describe a grading system for evaluating the severity of organ injuries associated with *Vibrio* spp. infection. Garnier and co-workers [17] reported necrotic lesions in different connective tissues and in the adductor muscle, and atrophy of the epithelium of the digestive gland. Additionally, Parizadeh et al. [6] described histologically localized bacteria in oyster tissues associated with lysis of the sub-epithelial connective tissue of the mantle, atrophy of the digestive gland, and infiltration in hemolymphatic vessels in oysters experimentally infected with *V. aestuarianus*. 

This study investigates the occurrence of the most common oyster pathogens (OsHV-1, *Vibrio* species) and related immune response in target organs during a mass mortality outbreak that occurred in Pacific oysters farmed in Sardinia by combining cultural, biomolecular, and histopathological methods. 

The potential contribution of *V. aestuarianus* to oyster mortality and the relevance of mantle hemocyte infiltration in *Vibrio* infection diagnosis are also discussed. 

## 2. Results

Oyster mortality episodes were recorded during the winter season from December 2016 to February 2017, when water temperatures ranged from 13 to 14 °C (Supplementary Table S1). Oysters (n = 358) were mostly found moribund during the aforementioned period.

Microbiological analyses from the digestive gland of individual oysters (n = 358) resulted in the isolation of 376 bacterial strains. Of 376 strains, 100 were biochemically identified as *Vibrio* spp. (>95% identity), while 276 were ascribed to *Vibrio* spp. low identity, or to environmental bacteria of uncertain identification.

Molecular identification of *Vibrio* spp. by toxR PCR conducted on the 376 bacterial strains (100 *Vibrio* spp. > 95% identity + 276 low identity *Vibrio* spp. and environmental bacteria) allowed to assign 71 (18.9%) strains to *V. aestuarianus* and 166 (44.1%) strains to *V. splendidus* (Table 1). One hundred thirty-nine strains were molecularly identified as environmental bacteria and were discarded for further analysis.

Histologically, 52 out of 71 (73.2%) *V. aestuarianus* toxR PCR positive digestive glands showed mild to severe hemocyte infiltration (Table 1), 18 digestive glands did not show hemocyte infiltration and 1 sample was not able to be evaluated by histology. Hemocyte infiltration was also observed in 215 out of 287 (74.9%) *V. aestuarianus* PCR negative digestive glands. Both *V. aestuarianus* PCR positive and negative digestive glands were more commonly associated with a mild intensity of infiltration (χ2 (2) = 6.4767; *p* < 0.05). Similarly, 126 out of 166 (75.9%) *V. splendidus* toxR PCR positive digestive glands were associated with mild to severe hemocyte infiltration (Table 1), 39 digestive glands did not show hemocyte infiltration, and 1 sample was not evaluable by histology. In the digestive glands, differences in hemocyte infiltration between PCR-positive and PCR-negative samples were not statistically significant (both in the case of *V. aestuarianus* and *V. splendidus*) (*p* > 0.05). 

Twenty-eight out of 358 (7.8%) gills and mantle pools (see materials and methods) were positive for *V. aestuarianus* by toxR PCR, while *V. splendidus* toxR PCR was negative in the same samples. 

Oyster PCR positives to *V. aestuarianus* were mostly observed during the mortality episode (Supplementary Table S1). Similar to that observed in the digestive gland, PCR-positive gills (n = 28) showed mild to severe hemocyte infiltration by histopathology (24 out of 28; 85.7%) (Table 1), two gills did not show hemocyte infiltration, and two gills were not able to be evaluated by histology. Hemocyte infiltration was also observed in 178 out of 330 (53.9%) PCR-negative samples. 

The difference in hemocyte infiltration between PCR positive and PCR negative gills was statistically significant (Fisher’s Exact test *p* < 0.05). By histopathology, *V. aestuarianus* PCR-positive gills showed mostly mild hemocyte infiltration, and more rarely moderate and severe infiltration (Table 1).

**Figure 1:**
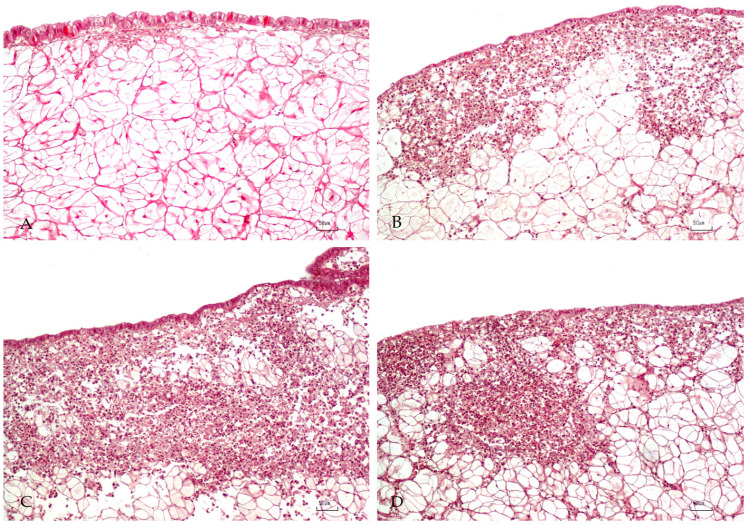
Oyster mantle. (**A**) normal tissue; (**B**) moderate and (**C**) severe hemocyte infiltration; (**D**) nodular distribution of hemocyte infiltrates. Bar A-B-C-D = 50 μm.

Histopathology revealed hemocyte infiltration in 100% (n = 28) of *V. aestuarianus* PCR-positive mantles, mostly moderate and severe (Table 1); of these, 12 samples showed hemocyte infiltration in hemolymphatic vessels. In particular, hemocytes were prevalently observed in sub-epithelial mantle connective tissue, and their distribution was multifocal, diffuse, or nodular [22,23] (Figure 1). Nodular aggregation of hemocytes was mostly observed in association with severe infiltration (Figure 1), and hemocytes were often located within hemolymphatic vessels. 

Hemocyte infiltration was also observed in 157/330 mantle PCR negative mantles (47.6%), and 19 mantles were not able to be evaluated by histology.

Hemocyte infiltration was more frequently observed in *V. aestuarianus* PCR-positive mantles compared to negative mantles (Fisher’s Exact test *p* < 0.01). Interestingly, positive mantles were most commonly associated with a moderate degree of hemocyte infiltration (78.6%), while mild infiltration was most frequently recorded in negative mantles (80.3%) (χ2 (1) = 54.8119; *p* < 0.01).

*V. aestuarianus* bacterial loads in gills and mantle pools ranged from 6.05 × 10^2^ copies/μL to 7.55 × 10^6^ copies/μL. Five out of 28 samples were 10^2^ copies/μL, 11 out of 28 were 10^3^ copies/μL, 7 out of 28 were 10^4^ copies/μL, 4 out of 28 were 10^5^ and 1 out of 28 samples had 10^6^ bacterial loads. Furthermore, the highest *V. aestuarianus* loads (*p* = 0.1) were found in pools associated with mantle sub-epithelial connective tissue with moderate and severe hemocyte infiltration.

Gills and mantle pools were negative for *V. splendidus* ToxR PCR (Table 1).

Notably, PCR failed to detect OsHV-1 in gills and mantle pools extracted from all the oysters examined (n = 358). 

PCR products (n = 28) obtained from gills and mantle pools with *V. aestuarianus* ToxR PCR were successfully cloned into pCR4-TOPO. Sequencing revealed an invariable sequence of 219 nucleotides and the strain was designated *V. aestuarianus* Sar1. BLASTN results are summarised in Table 2. Briefly, *V. aestuarianus* Sar1 shared 99% to 100% with several *V. aestuarianus* subsp. *francensis* strains isolated by Garnier and coworkers [17]. 

**Figure 2:**
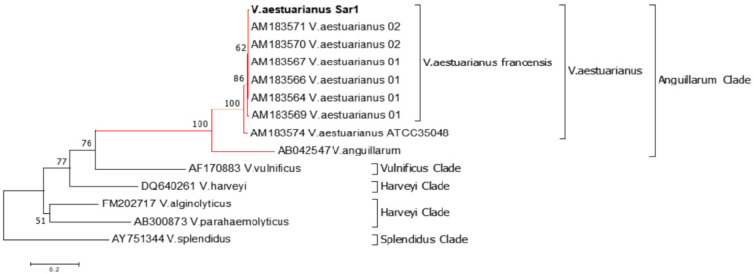
Recovery of oysters Vibrio clades and phylogeny of *V. aestuarianus* Sar1 based on the *toxR* gene. The evolutionary history was inferred using the neighbor-joining method. The optimal tree with the sum of branch length = 2.93743942 is shown. The percentage of replicate trees in which the associated taxa clustered together in the bootstrap test (1000 replicates) are shown next to the branches. The tree is drawn to scale, with branch lengths in the same units as those of the evolutionary distances used to infer the phylogenetic tree.

Phylogenetic analyses, based on the alignment of V. aestuarianus Sar1 toxR gene with 13 sequences representative of *Vibrio species* infecting oysters, were consistent with that previously observed with other phylogenetic probes (Figure 2). *V. aestuarianus* Sar1 is clustered in a monophiletic clade (*Anguillarum clade*) including *V. aestuarianus* francensis and *Vibrio anguillarum*. *Vulnificus*, *Harveyi*, and *Splenidus clades* are also recovered in the analysis. 

## 3. Discussion

Oyster pathogens such as OsHV-1 and *Vibrio* spp. are ubiquitous marine agents associated with *Crassostrea gigas* severe mortalities in the field [17,20,21,24,25]. Among *Vibrio* species pathogenic to oysters, *V. aestuarianus* has been commonly reported in mass mortality episodes, often in coinfections with OsHV-1, worldwide [6,10]. Recently, *Vibrio aestuarianus* in association with *Tenacibaculum soleae* have been associated with the oyster mortality outbreak in the San Teodoro lagoon [3].

Here we investigated and tentatively discuss the role of *Vibrio aestuarianus*, *Vibrio splendidus*, and OsHV-1 in a mortality outbreak that occurred in the San Teodoro lagoon. 

Initially, the presence of OsHV-1 was excluded by PCR in oyster gills and mantle, the most common target tissues for molecular investigations. In turn, in the oysters’ digestive glands, microbiological isolation combined with ToxR PCR demonstrated the presence of *Vibrio aestuarianus* and *Vibrio splendidus*. Notably, the involvement of *Vibrio* species other than *Vibrio aestuarianus* and *Vibrio splendidus* was ruled out by the use of dedicated PCR tests conducted on the oyster digestive gland (data not shown). These results are consistent with what was previously reported by several authors, finding *Vibrio* spp. as the most common bacteria in the digestive gland of oysters [26]. 

In digestive glands, hemocyte infiltration was equally observed in *V. aestuarianus* negative PCR samples compared to positive samples; moreover, both positive and negative samples were more commonly associated with a mild intensity of infiltration. The same was observed in the case of *V. splendidus*. These findings suggest that the proliferation of immune cells in the oyster’s digestive gland is not only related to the presence of *V. aestuarianus* and *V. splendidus*. It has been suggested that the presence of immune cells in the oyster digestive gland could be physiological, because of the functions of this organ and its continuous interaction with pathogenic and non-pathogenic microbial agents [27,28]. Consistent with reports by other authors [29], beside *Vibrio* spp. we detected metazoan parasites associated with a mild to moderate hemocyte infiltration in the oyster’s digestive gland (data not shown). Based on these findings we postulate that the oyster digestive gland does not represent the ideal target organ to investigate the specific role of a given pathogen during mortality episodes.

The oyster gills and mantle are also common target tissues for investigating the presence of *Vibrio* spp., both in the field and under experimental infection [6,21]. In this study, *V. aestuarianus* was detected by PCR in 7.8% gills/mantle pools, whereas the presence of *V. splendidus* in the same tissues was ruled out. 

In gills, histology showed hemocyte infiltration in 85% *V. aestuarianus* PCR positive samples, mostly with a mild degree of severity (57.1%). A statistically significant difference was observed when comparing hemocyte infiltration in PCR positive with PCR negative samples. Tentatively, hemocyte infiltration observed in PCR negative samples could be explained by the presence of parasites observed by histopathology in the same tissues (data not shown). Also, gills are filtering organs continuously exposed to microbial agents [28].

In the mantle, hemocyte infiltration was detected in 100% of *V. aestuarianus* PCR positive samples, mostly showing moderate to severe hemocyte infiltration. Conversely, *V. aestuarianus* PCR negative samples were mostly associated with mild hemocyte infiltration. Interestingly, highest *V. aestuarianus* loads were found in oysters showing moderate to severe hemocyte infiltration in mantle sub-epithelial connective tissue. Additionally, in *V. aestuarianus* positive samples, moderate to severe hemocytic infiltration was also observed around and within hemolymphatic vessels. This is consistent with what was reported by Parizadeh et al. [6] during an experimental infection. Therefore, we postulate that *V. aestuarianus* alone could be associated with the severity of hemocyte infiltration in an oyster mantle. This seems to be confirmed by histopathology, which showed the absence of parasites in the same tissues. However, further investigations based on a greater number of oysters and demonstrating the colocalization of bacteria and immune cells in damaged tissues are required to confirm this hypothesis. 

Limited information is available about host colonization, transmission, pathogenicity of *Vibrio* spp. in oysters. The ability of *Vibrio* spp. to induce disease seems to be strongly affected by environmental factors, (i.e., temperature, salinity, and physiological factors) [10,30,31]. 

We report a mortality outbreak in San Teodoro lagoon during the winter season (December 2016 to February 2017) causing the final loss of more than 80% of farmed oysters. During this season the lowest water temperatures of the year were recorded (13 to 14 °C). This, according to other authors [3,6,10], reinforces the hypothesis that interconnected factors (pathogenic bacteria and environmental factors) may act together in synergy inducing mortality episodes. However, the influence of temperature on *V. aestuarianus* and its ability to infect oysters remains underinvestigated. 

Interestingly, PCR positives to *V. aestuarianus* overlapped oyster mortality’s time span, and this observation, together with the absence of PCR positives to OsHV-1 and *V. splendidus* in the same tissues reinforce the hypothesis that *V. aestuarianus* could act as the most relevant pathogen causing mortality.

The genetic similarity of *Vibrio aestuarianus* Sar1, as identified in this study, to *Vibrio aestuarianus* subspecies *francensis* strains associated with different percentages of oyster mortality could be a further indication of the pathogenicity of *Vibrio aestuarianus* Sar1 and suggest a continuous monitoring of the San Teodoro lagoon farm. Phylogeny-based on the ToxR gene proved useful to assign *Vibrio aestuarianus* Sar1 to the Anguillarum cluster, and to recover other clusters of *Vibrio* species infecting oysters. However, evolutionary studies based on the full ToxR gene are needed to evaluate the full potential of this phylogenetic probe. 

## 4. Materials and Methods 

### 4.1. Samples

Three hundred and fifty-eight samples of juvenile and adult cupped oysters, *Crassostrea gigas*, were collected from October 2016 to June 2018 in the San Teodoro lagoon, one of the most devoted areas for oyster farming in Sardinia (Italy). Oysters were collected during 18 monthly samplings, during which water temperature and salinity were recorded. 

Oysters were sampled directly from poches and immediately transported within refrigerated containers to the laboratory, where gross examination was carried out in order to verify the physiological status and vitality of oysters. 

Digestive glands of each specimen were specularly sectioned for bacterial isolation, PCR extraction, and histopathological examination. 

A portion of gills and a portion of mantle of each specimen were pooled for DNA extraction, while a portion of the same tissue was individually formalin-fixed for histopathology. 

### 4.2. Bacteriological Analysis

For microbiological isolation, digestive glands of individual oysters were sampled with a sterile inoculation loop in aseptic conditions and seeded in brain heart infusion agar (BHI agar, Difco), supplemented with 1.5% NaCl. After a 24 to 48 h incubation at 17 to 20 °C. Bacterial strains were purified with subcultures on BHI agar until a pure culture was obtained [32]. 

Bacterial strains were identified using a polyphasic approach including firstly morphological and biochemical investigations and subsequently biomolecular analysis.

Morphological evaluation was microscopically performed by Gram staining of bacterial colonies using a commercial kit (Becton Dickinson, Le Pont-De-Claix, France). This approach allowed for the selection of *Vibrio*-like strains (small Gram-negative bacteria, rod shape) from environmental strains such as big Gram-negative rod bacteria typical of marine and brackish waters or Gram-negative sphere bacteria. The bacterial strains, of which the morphology or staining characteristics did not correspond with our study on oyster bacterial pathogens, were discarded.

The biochemical profile of *Vibrio*-like isolated strains was determined by using SIM, O/F (with filtered seawater), oxidase and catalase tests, and the API 20 NE identification system [17]. The primary tests for the determination of biochemical characteristics were performed according to the Manual of Methods for General Bacteriology (American Society for Microbiology, 1981). These biochemical tests allowed us to distinguish *Vibrio* spp. from other environmental strains.

### 4.3. DNA Extractions, Diagnostic PCRs, and Quantitative PCR

The mantle and gills were collected from each specimen (n = 358) and individually pooled. Total DNA was extracted from the 358 pools by using the QIAamp DNA mini kit (Qiagen GmBH, Hilden, Germany), according to manufacturer instructions. DNA was also extracted from the 376 strains isolated from the digestive gland by using the Nexttec DNA isolation kit for Bacteria (Nexttec GmbH, Leverkusen, Germany), according to the manufacturer’s instructions.

DNA samples obtained from mantle and gill pools were tested by PCR in order to investigate the presence of OsHV-1. PCR was performed as previously described [33] by using C2 and C6 primers, with minor modifications. Briefly, PCR reactions were carried out using an MJ Mini Thermal Cycler (BioRad, Hercules, CA, USA); 25 μL PCR reaction mix volume contained 1X reaction buffer, 3 mM MgCl_2_, 2 mM of each dNTP, 2 U/μL Taq polymerase (Life Technologies, Camarillo, CA, USA), 0.2 pmol/μL of each primer (MWG-Biotech, Ebersberg, Germany), 1 μL of extracted DNA and distilled water. PCR thermal conditions were set as follows: 1 cycle of initial denaturation at 94 °C for 2 min; denaturation at 94 °C for 30 s, primer annealing at 54 °C for 30 s and elongation at 72 °C for 1 min, the last 3 steps were repeated 35 times; finally, the last step was 1 cycle of final elongation at 72 °C for 7 min. Two percent agarose gel electrophoresis (Sigma-Aldrich, St. Louis, MO, USA) with 10,000X SYBR Safe as DNA staining (Invitrogen, Carlsbad, CA, USA), Blue Loading Buffer (Invitrogen) and 100 bp DNA Ladder (Invitrogen) and a transilluminator Safe Imager were used to highlight amplified fragments. Images were acquired by the Photodoc system (Invitrogen).

PCR was also used to identify bacterial isolates obtained from the oyster digestive gland, and to verify the presence of *V. aestuarianus* and *V. splendidus* in the 358 gills and mantle pools. With this aim, primers were designed to amplify a 259 bp fragment of the *V. aestuarianus* gene encoding the *toxR* transmembrane regulatory protein (VesToxF 5′ CAAAGAACCGGTGGTCGAGC 3′; VesToxR 5′ ATTGTAGACAGCCAATTGCC 3′). Similarly, primers were developed for *V. splendidus* (VspToxF 5′ TTTGCAACGCCTACAATGAC 3′; VspToxR 5′ ATTGGCATGATGAAAGCCGC 3′). In order to amplify both *V. aestuarianus* and *V. splendidus* from the gills and mantle the following PCR protocol was developed: PCR reactions (25 µL) contained 1 mM MgCl_2_, 3 µL of Mastermix (Larova), 0.2 µM of each primer, 1 µL of extracted DNA; cycling included 1 cycle of initial denaturation at 94 °C for 2 min, 30 cycles of denaturation at 94 °C for 20”, primer annealing at 55 °C for 20”, and elongation at 72 °C for 20”; final elongation at 72 °C for 10 min. 

Quantitative real-time PCR was performed on DNA extracted from gill and mantle tissue pools in order to quantify *V. aestuarianus* positive samples. A PCR reaction was carried out with 25 µL reaction mix containing 1.5 mM MgCl_2_, 15 µL of Crystal Taq Master (Larova), 1 µL Sybr Green, 0.2 µM of each primer 1 µL of extracted DNA and distilled water; it was performed by using Rotor-Gene Q (Qiagen GmbH, Germany). Thermal conditions were: 1 cycle of initial denaturation at 95 °C for 2 min; denaturation at 95 °C for 10 s, primer annealing at 60 °C for 15 s and extension at 72 °C for 60 s, the last 3 steps were repeated 40 times; the melting curve was performed at 95 °C, 45 °C, and 95 °C, and the temperature increased by increments of 1 °C, waiting for 5 s before each acquisition. Finally, the system cooled down to 35 °C. The quantification of the samples was based on a standard curve with five dilution points from 1.5 × 10^8^ copies/µL stock of genomic DNA extracted from a bacterial suspension of the reference strain (*V. aestuarianus* DSM 19606), used as the positive control. Real-time PCR assay correlations with coefficient values (r2) of >0.97 and reaction efficiency of >0.90 were accepted in this study [24,34].

### 4.4. Sequencing and Post-Amplification Analysis

PCR products obtained with primers VesToxF and VesToxR were cloned into the plasmid pCR4-TOPO (Life Technologies, CA). Ligation products were subsequently used to transform One ShotTOP10 Chemically Competent Escherichia coli (Invitrogen, Monza, Italy). Plasmid DNA was extracted with PureLink Quick Plasmid MiniPrep Kit (Invitrogen, Monza, Italy), and automatically sequenced (BMR Genomics, Padova, Italy). Sequences were edited with 4Peaks V 1.8 (Technelysium, Amsterdam, The Netherlands), and aligned with ClustalW [35] to assign them to unique sequence types. The unique sequence type obtained in this study was named *V. aestuarianus* Sar1, and it was checked against the GenBank database using BLASTN [36]. *V. aestuarianus* Sar1 sequence was deposited in the GenBank under accession number MT472290. 

Phylogenetic analyses were performed by aligning *V. aestuarianus* Sar1 with a set of sequences belonging to 13 Vibrio species that infect oysters. Evolutionary history was inferred using the neighbor–joining method [37] implemented in MEGA7 [38]. All positions containing gaps and missing data were eliminated. There was a total of 203 positions in the final dataset. The robustness of trees was evaluated by bootstrapping with 1000 replicates [39]. Evolutionary distances were computed using the Jukes–Cantor method [40] and were in units of the number of base substitutions per site.

### 4.5. Histological Analysis

A standard section of the oysters containing target tissues (digestive gland, gills, and mantle) was fixed in 10% neutral buffered formalin; sections were dehydrated through alcohol and xylene, using an automatic tissue processor, and paraffin-embedded, according to standard techniques [41]. Sections 3 μm thick were stained with Hematoxylin and Eosin (H&E) and examined with a light microscope. A Gram stain on histological sections was also performed to provide evidence of the presence of bacteria.

### 4.6. Statistical Analysis

Fisher’s exact or Chi-square (χ2) tests were used to determine the association between histopathological and biomolecular results. Accordingly, quantitative real-time PCR values range from 10^2^ to 10^3^ copies/μL and greater than 10^4^ copies/μL were categorized as low and high bacterial loads, respectively. All the statistical analyses, including descriptive statistics, were performed using Stata 11.2 software (StataCorp LP, College Station, TX, USA). Statistical significance was defined as *p* < 0.05. 

## 5. Conclusions

In the Mediterranean area, oyster aquaculture is increasingly gaining economic importance. Whereas several microbial species have been identified causing oyster mortality, the association of *V. aestuarianus* and mortality outbreaks is still poorly investigated in the field. Currently, the main methods for the detection of oyster pathogens are based on biomolecular and microbiological techniques. Bivalve histopathology has recently become a crucial tool for disease diagnosis of aquatic organisms, albeit only a few studies have focused on the importance of histopathological investigation to detect the presence of inflammatory reaction, and tissue lesions associated to mollusk pathogens [6,21,42,43,44,45,46,47].

This study represents an effort in this direction, by combining molecular tools with histopathological investigation on farmed oysters. This integrated approach revealed a statistical association between hemocyte infiltration in the mantle and *V. aestuarianus* bacterial loads and provides an indication of a potential role for *V. aestuarianus* in this oyster mortality. However, further analyses, based on a larger number of samples and on in situ hybridization and immunohistochemistry, are recommended to localize *V. aestuarianus* in tissues and to fully establish the correlation between this pathogen and mantle damage. To conclude, results pave the way to the development of tools for the control and management of farmed oysters and provide information useful for clarifying the role of *V. aestuarianus* in oyster mortality.

## Figures and Tables

**Table 1:** Histopathological evaluation of hemocyte infiltration in the digestive gland, gills, and mantle of oysters in PCR positive samples for *V. aestuarianus* and *V. splendidus*.

PCR +			* H. I.				
					Mild	Moderate	Severe
Digestive gland	*V. aestuarianus*	71/376	*V. aestuarianus*	52/71	33 (46.5%)	15 (21.1%)	4 (5.6%)
	*V. splendidus*	166/376	*V. splendidus*	126/166	88 (53%)	33 (19.9%)	5 (3%)
Gill and mantle individual pools	*V. aestuarianus*	28/358	gills	24/28	16 (57.1%)	7 (25%)	1 (3.6%)
	*V. splendidus*	0/358	mantles	28/28	3 (10.7%)	22 (78.6%)	3 (10.7%)
					-	-	-

* H. I. = hemocyte infiltration at histology.

**Table 2:** Results of BLASTN analysis.

	*V. aestuarianus* subsp. *francensis* Strains		
	02/114; 02/103;02/041; 01/140; 01/064; 01/031	02/093	01/308
*V. aestuarianus* **Sar1**	100%	99%	99%

## Supplementary Materials

The following are available online at https://www.mdpi.com/2076-0817/9/6/492/s1, Table S1: San Teodoro lagoon: number of oysters PCR-positive for *V. aestuarianus* during mortality outbreaks associated with water temperature and salinity.
