# Supplementary material for: Histopathological and Molecular Study of Pacific Oyster Tissues Provides Insights into V. aestuarianus Infection Related to Oyster Mortality
Source: Pathogens. 2020 Jun 20;9(6):492. doi: 10.3390/pathogens9060492 (PMC7350300; doi:10.3390/pathogens9060492)
Supplement: Supplementary file 1 [file pathogens-09-00492-s001.zip › pathogens-831171-SI.pdf]

Table S1. San Teodoro lagoon: number of oysters PCR positive to *V. aestuarianus* during mortality outbreaks associated to water temperature and salinity.

Sampling Date	N° Samples	*V. aestuarianus* PCR Positive (Gills/Mantle Pools)	Water Temperature	Water Salinity
October 2016	23	-	22 °C	40‰
November 2016	16	-	16 °C	40‰
**December 2016** *	30	14	14 °C	40‰
**January 2017** *	24	3	13 °C	40‰
**February 2017** *	24	6	14 °C	10‰
March 2017	24	-	16 °C	36‰
April 17	21	-	18 °C	42‰
May 2017	18	-	20 °C	40‰
June 2017	22	-	28 °C	40‰
July 2017	20	-	25.3 °C	41‰
August 2017	16	-	26 °C	42‰
September 2017	26	-	22 °C	42‰
October 2017	18	-	18.5 °C	44‰
November 2017	15	-	17.5 °C	40‰
December 2017	12	-	12 °C	42‰
January 2018	18	-	13 °C	24‰
February 2018	14	-	13 °C	36‰
June 2018	17	5	24.4 °C	40‰
Total	358	28		

* Mortality outbreaks time span.
